# The Effects of Data-based Instruction (DBI) for Students with Learning Difficulties in Korea: A Single-subject Meta-analysis

**DOI:** 10.1371/journal.pone.0261120

**Published:** 2021-12-23

**Authors:** Dongil Kim, Seohyeon Choi

**Affiliations:** 1 Department of Education, Seoul National University, Seoul, Korea; 2 Department of Educational Psychology, University of Minnesota, Minneapolis, MN, United States of America; University of Eastern Finland: Ita-Suomen yliopisto, FINLAND

## Abstract

Data-based instruction (DBI) is an ongoing process to utilize students’ data for determining when and how to intensify intervention. It is an educational approach that is suggested as effective to enhance achievements of struggling learners, particularly for those who did not respond to intensive intervention in usual ways. In Korea, DBI was introduced and applied for students with learning difficulties especially since 2000 when the first Korea curriculum-based measurement (CBM) was developed as the name of Basic Academic Skills Assessment. Despite a number of studies accumulated since then, there has been a lack of research that examined the level of evidence-based practice (EBP) of DBI research. Thus, the present study sought to synthesize the DBI research so far in Korea by analyzing the effectiveness of DBI for school-aged students with learning difficulties via meta-analysis and evaluating the quality of the research. In this study, a total of 32 single-subject design studies were used. Multilevel meta-analysis revealed that the mean effect size of DBI was statistically significant (*B* = 1.34) and there was significant variance across participants in effect sizes. The results from the conditional model showed that exceptionality type, the number of sessions, and the length of each session were significantly accountable for the variability of effect sizes. In addition, the results of the qualitative analysis revealed the acceptable quality of the overall DBI research with some limitations. Based on these findings, implications and study limitations were discussed.

## Introduction

In general school classrooms, there are groups of students who have severe difficulties in acquiring and using basic learning skills. These are very diverse and heterogeneous groups, and may be affected by a variety of factors, including learning disabilities, dyslexia, emotional or behavioral problems, below-average or borderline intelligence, multicultural backgrounds, environmental deficits, and insufficient educational opportunities. The term ‘learning difficulties’ refers to the large group of students who exhibit severe problems with learning and need extra assistance with schooling [1]. This generic term reflects the willingness to provide proper intervention and educational services to diverse students with or without internal causes, who exhibit severe difficulties in basic academic areas. It is also related to the OECD model of students with “special educational needs”, which encompasses disability, difficulties and social disadvantage [2, 3]. In this comprehensive and resource-based approach, heterogeneous groups of students can be inclusively involved [3], and they can be served before referral to special education eligibility.

Data-based instruction (DBI) is suggested as an effective approach to enhance academic outcomes of students with severe learning difficulties. DBI is also called ‘data-based instruction’ [4] or ‘data-based individualization’ [5]. This concept originated from the concepts of ‘data-based program modification’ suggested by the study of Deno & Mirkin [6] and ‘problem solving model’ in 1980s, which was used by Minneapolis Public Schools (MPS) as an alternative method of educational decision-making for learning disabilities and mild disabilities [7]. DBI is defined as a series of successive and systematic procedures in reading, mathematics, or behavior where students’ data of achievement are used to determine when and how to intensify and modify interventions [5].

To be specific, the procedures of DBI involve the following steps: (a) identifying a student’s current level of performance, (b) establishing a long-term academic goal, (c) implementing quality evidence-based intervention with fidelity while monitoring one’s progress frequently, (d) using data-based decision rules in order to determine whether instructional changes would be needed, (e) establishing a tentative hypothesis about the student’s specific needs and implementing changes in instruction based on the previous hypotheses, (f) evaluating the effectiveness of the instructional changes based on progress monitoring data, and (g) repeating these procedures until the student achieves the academic goal [8, 9]. A strong evidence base supports the efficacy of DBI for students with learning difficulties [10–12]. Moreover, DBI has positive influence on teachers’ instructional planning, leading them to make more specific plans, to make instructional adaptations more frequently, and to identify the targeted skills more appropriately [13, 14].

It is important to use a reliable measurement that is sensitive to the struggling students’ growth over a short period of time in order to make ongoing decision in DBI [8]. In this purpose, curriculum-based measurement (CBM) is used to decide whether to raise the goal of instruction, keep instruction as-is, or change instruction [15]. CBM, which was first developed by Stanley Deno and his colleagues at the University of Minnesota, is a standardized measurement for assessing students’ academic competence and progress in the basic academic domains [16]. It is inexpensive and efficient to implement and has high adequacy in terms of validity and reliability [17]. Moreover, as a ‘general outcome measure’, the scores of CBM are considered to represent an individual’s generalized level of the corresponding domain [18].

In Korea, the concept of DBI was introduced and has been applied for teaching school-aged students with learning difficulties, especially since 2000 when the Korean CBM was developed as the name of Basic Academic Skills Assessment (BASA) [19, 20]. According to the study of Yeo, Hong, & Son [21] who reviewed the trend of CBM research in Korea from 1999 to 2014, BASA was found to be the most widely used CBM tool in Korea. So far, BASA has been developed in eight basic academic areas: reading, writing, mathematics, math word problems, vocabulary, reading comprehension, early mathematics, and early literacy. Since BASA includes a number of equivalent tests, it can be conducted at regular basis for progress monitoring in the process of DBI. For data-based decision rules, it recommends using the data-point method (i.e., to make instructional change when more than 4 successive scores below or more than 3 successive scores above the target line) and the slope-based method (i.e., to compare the slope of the progress line with the slope of the target line).

In educational practice, general and special teachers are required to use interventions which were scientifically proven. In the field of special education, there have been constant efforts to identify and implement evidence-based practices [22]. In order to examine whether the specific intervention and the studies that implemented it have some scientific evidence, it is first necessary to evaluate the quality of the individual studies in terms of study design and method [23]. For evaluating the quality of studies in special education field, the quality indicators (QIs) for four types of research methodologies (i.e., group experimental, correlational, single subject, and qualitative designs) were developed by the task force team at the Council for Exceptional Children’s (CEC) [23]. Specifically, Gersten et al. [24] identified QIs for group experimental and quasi-experimental studies, and Horner et al. [25] identified QIs for single-subject designs. Second, it should be proved in a number of studies that the effectiveness of the intervention is considerably large. In this purpose, meta-analysis is widely used to statistically synthesize the results from more than two separate studies.

In the present study, it aims to analyze the overall effectiveness of DBI for school-aged students with learning difficulties and the quality of research in Korea. The present study attempts to collect and synthesize only single-subject design studies. Specifically, it examines the quality of the studies using the QIs suggested by Horner et al. [25]. In addition, it synthesizes the effectiveness of those interventions via meta-analysis and identifies the potential variables to explain the variances of the effect sizes. As of now in Korea, there is a lack of research that specifically synthesizes the effectiveness of DBI. Although Yeo, Hong, & Son [21] synthesized CBM research in Korea, their research questions were focused on CBM and the aspects of technical adequacy. Moreover, it was not a meta-analytic review but a narrative one. Another literature review conducted by Jung [26] was focused on the theme of DBI, but only international studies were included. Therefore, the present study poses the following research questions:

*RQ1*: What are the characteristics and quality of studies on DBI implemented for students with learning difficulties?
*RQ2*: What is the average effect of DBI on basic academic skills for students with learning difficulties?
*RQ3*: To what extent have participant-, intervention-, DBI-related variables influence on the effectiveness of DBI for students with learning difficulties?

## Methods

### Search procedures

A comprehensive literature search was conducted using the Korean electronic databases RISS (Research Information Sharing Service), KISS (Korean studies Information Service System), and Nurimedia DBpia in order to retrieve studies that are written in Korean and published until December 1, 2020. Since the number of research on DBI for students with learning difficulties was not large, no restriction was set regarding dates of publication. For this search, keywords for students with learning difficulties (“learning difficulties”, “learning disabilities”, “at-risk”, “low achievement”, and “underachievement”), keywords for basic academic skills (“basic academic”, “basic learning”, “reading”, “writing”, and “math”), keywords related to DBI (“data-based instruction”, “evidence-based instruction”, “data-based individualization”, and “curriculum-based measurement”), and keywords for BASA (“Basic Academic Skills Assessment”, and “BASA”) were used in various combinations, and Korean terms were used for the search. Furthermore, reference lists both from prior syntheses and the included studies were reviewed to encompass any possible studies to be analyzed. By including the keywords for BASA, it reduced the likelihood to miss the studies that did not use the term “DBI” explicitly but were grounded on DBI using BASA. This database search yielded 422 studies after duplicates removed. From these, a total of 390 studies were excluded from the final analyses based on the following inclusion and exclusion criteria.

### Inclusion and exclusion criteria

**Fig 1:**
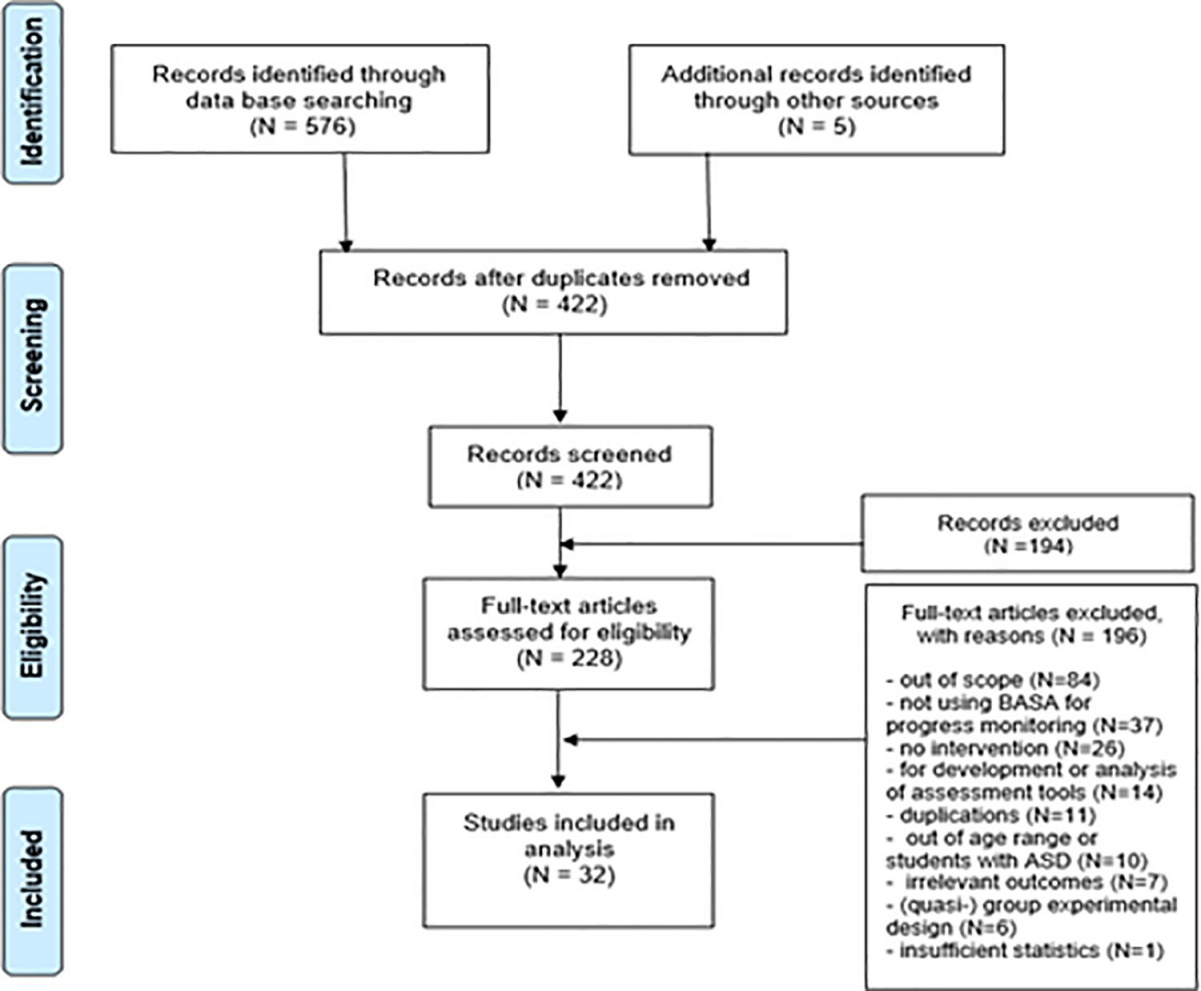
PRISMA flowchart for data collection.

Among the preselected studies, only studies which met all the following criteria and had full text accessible were included in the analysis. First, studies with students with learning difficulties as participants were included. Terms such as “students with/at-risk of learning disabilities”, “struggling learners”, “low achievers”, and “under-achievers” were considered to fall within the category of students with learning difficulties. Studies for participants with intellectual disabilities were included in this study. Second, participants were school-aged children from six to eighteen. Third, studies that implemented intervention on basic academic skills (reading, writing, and math) and had dependent variables for those skills were included. There was no restriction in the type of intervention as long as basic academic skills were set as dependent variables. In several studies of which aims were beyond to determine the effectiveness of intervention (e.g., latent class analysis of struggling learners, examination of the applicability of RTI approach, or screening of students with learning disabilities), they were accepted when they had implemented the procedures of DBI on basic academic skills. Fourth, studies that conducted intervention following the principal rules of DBI (i.e., frequent progress monitoring and the use of data for ongoing instructional decision making) were included. Fifth, only the studies that used BASA as CBM tools were included for the analysis, which is the standardized and most frequently used monitoring tool and has high technical adequacy. Other researcher- and teacher-generated CBM were excluded, as they had been pointed out to have a lack of evidence of validity and reliability [8, 21]. Sixth, studies of single-subject designs providing enough quantitative statistics to calculate effect sizes were accepted. Fig 1 below summarizes these procedures of literature selection according to the PRISMA 4-phase flow diagram.

### Coding procedures

#### Coding system for quality indicators

QIs suggested by Horner et al. [25] were used to examine each study. However, it has been pointed out in the previous studies that these QIs have strict criteria and that evaluating in a dichotomous way is quite difficult [27, 28]. Thus, while the present study is primarily based on the QIs of Horner et al. [25], it evaluated the quality level of each indicator in a continuum using the 3-point Likert scale. For QI rating, it referred to the rubric suggested by Chard et al. [29] which was based on 4-point Likert scale. The individual study was interpreted as having a sufficient quality level when the average is above 2 points in each area.

For the inter-rater reliability of the qualitative analysis, in addition to the authors, a doctoral student majoring in special education who had understanding of QIs participated in the analysis. Two from single-subject design studies were randomly selected. For those studies, we rated together on a 3-point Likert scale, ensuring that we had the same understanding of the concept and evaluation standard. After that, the authors of the study conducted the analysis of QIs for 32 studies first. Then, the doctoral student reviewed the whole analysis results and identified the items that were rated differently. For the inconsistent items, we conducted an additional review and reached to the consensus through sufficient discussion. The reliability between two coders was 99.6%, which was calculated by dividing the number of consistent results by the sum of the number of both consistent and inconsistent results and multiplying it by 100.

#### Coding system for meta-analysis

A coding system for meta-analysis was developed by examining the prior coding systems designed for meta-analytic review on DBI [8, 30] as well as research on DBI concepts [5, 10, 26, 31]. The coding system for meta-analysis is presented in Table 1.

**Table 1:** Coding system for meta-analysis.

Definition of Descriptors	
**General Characteristics**	
Year of publication	the publication year of study
Type of publication	the type of publication, recorded as (1) journal article (2) dissertation
**Participants**	
Grade level	the grade level, recorded as (1) elementary school (2) middle school (3) high school
Sample size	the number of participants
Gender	the number of male and female participants
Exceptionality type	the disability or difficulties participants have, recorded as (1) with or at risk of learning disability (2) low achievement (3) under-achievement (4) intellectual disability (5) other
**Intervention**	
Topic of intervention	the main topic of intervention within academic domains (e.g., intervention on math using play activities)
Instructional approaches	the types of instructional approaches (e.g., direct instruction)
Setting	the type of instructional setting, recorded as (1) general classroom (2) special education classroom (3) special school (4) private center (e.g., treatment center, hospital) (5) other (6) NA
Number of sessions	the total number of sessions
Length of each session	the length of each session (minutes)
Group size	the number of group members for instruction, recorded as (1) one-on-one (2) small (2–3) (3) medium (4–6) (4) NA
Interventionist	who provided the intervention, recorded as (1) researcher (2) teacher (3) undergraduate or graduate students (4) other (5) NA
Basic academic areas	the targeted basic academic domains (i.e., dependent variable), recorded as (1) reading (2) writing (3) math (4) other
**DBI**	
Type of CBM task	the type of CBM tasks (i.e., BASA) (e.g., reading fluency, early literacy, writing, early math, math)
Measurement frequency	the frequency of measurement with CBM for progress monitoring, recorded as (1) every session (2) every 2∼3 sessions (3) every 4 sessions (4) other
Administrator of CBM	who administered CBM, recorded as (1) DBI instructor (2) other (3) NA
Data-based decision rule	the type of decision rule for instructional adaptation based on data from progress-monitoring, recorded as (1) slope-based rule (2) point-based rule (3) criterion mastery (4) other (5) NA
Instructional adaptation	the type of instructional adaptation, recorded as (1) quantitative changes (e.g., group size, time) (2) qualitative changes (e.g., instructional strategies, arrangement of environmental variables, type of feedback) (3) combination (4) NA

For the reliability of coding, the doctoral student in addition to the authors who had research experiences using meta-analysis was participated in coding for the meta-analysis as well. The authors specifically explained the coding system and the coding method. Afterwards, three studies were randomly selected and coded by the coders together. Then, the doctoral student reviewed the results that were firstly coded by the authors, checking for the disagreed items. The coders went through the process of additional review and sufficient discussion regarding the inconsistent items, resulting in consensus at the end. Through these procedures, the reliability of coding for meta-analysis was calculated as 98.86%.

#### Effect size calculations and statistical analyses

For 32 single-subject studies, the effect size calculation was conducted through the following procedures. First, the data points of each dependent variable were coded. When the study only contains the figures for outcome measures, a computer software program GetData Graph Digitizer (2013) was used to extract graphed data. Second, the standardized data points and the effect sizes were calculated using the method which had been suggested by Van den Noortgate & Onghena [32–34] and used in Wang, Cui, & Parrila [35], Wang, Parrila, & Cui [36], and Heyvaert et al. [37]. To be specific, in order to transform raw scores into standardized scores, the raw scores of the data points were subtracted from the mean scores of the data points within the baseline and intervention phases and then divided by the standardized deviation of the data points from the phases combined [35]. Then, the standardized mean score of the baseline phase was subtracted from each standardized score, which was to adjust the mean of the baseline to 0. In this way, the standardized score of the intervention phase could be considered as the treatment effect. However, it should be noted that the standardized effect size calculated in this way cannot be directly compared to those calculated from group experimental design studies due to the difference in calculation method [35].

To synthesize the effect sizes calculated from single-subject studies, multi-level meta-analysis via hierarchical linear modeling was implemented. Meta-analysis has been increasingly being applied to single-subject design as well [38]. There is no consensus yet about what is the best statistical method or effect size indices when synthesizing the results from single-subject design studies [39]. However, it was pointed out that synthesis of results from single-subject studies tends to depend on visual analysis [32, 40], which was probably subjective and difficult to compare the effectiveness across different [35]. On the other hand, meta-analysis using hierarchical linear modeling is another promising approach [34, 41]. It can consider the issue of autocorrelation even when the number of measurements is relatively small and when the frequency of measurements is different across study participants [34, 42]. Before conducting the statistical analyses using hierarchical linear modeling, the normality assumption and the homogeneity assumption were tested based on the standardized scores. The Q-Q normality plot indicated that while some values deviate from the diagonal, most values do not appear to deviate significantly from what are expected for a random sample from a true normal distribution. The variance of the standardized scores across the phases and the participants were analyzed using the boxplots. The results revealed that there exists some variability across individuals especially for the baseline phases, but in overall, the medians and interquartile ranges are relatively consistent, indicating that the model meets the assumption of homogeneity of variance.

Statistical analyses were computed with HLM software program version 6 [43]. To be specific, the overall effect size and the heterogeneity of effect sizes between study participants were examined. In single-subject studies, each study includes different study participants, and each participant includes time series measurements, which represents the three-level model [44]. However, in the present study, the total number of single-subject studies was not enough to conduct a 3-level analysis. Thus, a 2-level meta-analysis was conducted. Level 1 represents the time series data points, and level 2 represents the participants of individual studies (see the following):

Level-1 Model: $Y_{ij}=\pi_{1j}(PHASE)+\epsilon_{ij}\;\epsilon_{ij}∼N(0,\sigma^{2})$
Level-2 Model: $\pi_{1j}=\beta_{10}+r_{1j}\;r_{1j}∼N(0,\tau_{oo}^{2})$

The two levels represent the hierarchical structure of outcome variables (level 1) and study participants (level 2). Level-1 model shows a regression equation for the outcome variable. In the model, the outcome variable, *Y*_*ij*_, represents the standardized score of the data point for occasion *i* and participant *j*; *π*_1*j*_ represents the effect size that equals the standardized difference of means between the intervention and baseline; *PHASE* is a dichotomous variable that represents the phase of each data point (i.e., 0 indicates that the data point is from the baseline phase and 1 indicates that the data point is from the intervention phase); *ϵ*_*ij*_ reflects a random error term. Level-2 model suggests that the effect size of its corresponding participant, *π*_1*j*_, equals the mean effect size across all participants (*β*_10_) plus a residual (*γ*_1*j*_). Furthermore, an additional analysis was conducted corresponding to each basic academic domain of the outcome variables (i.e., reading, writing, and math).

After analyzing the mean effect sizes across study participants with the above unconditional model, several variables related to participants, intervention, and DBI were added to the model as predictors in order to examine their impacts on the effectiveness. These predictors were added respectively, which was to avoid the potential risk of multicollinearity [37]. An instance of the conditional model is as the following.

Level-1 Model: $Y_{ij}=\pi_{1j}(PHASE)+\epsilon_{ij}\;\epsilon_{ij}∼N(0,\sigma^{2})$
Level-2 Model: $\pi_{1j}=\beta_{10}+\beta_{11}(\mathrm{GRADE})+r_{1j}\;r_{1j}∼N(0,\tau_{00}^{2})$

Additionally, there were several things considered in data analysis. First, when the intervention consisted of more than one phase, data from the very first intervention phase was coded to minimize the potential effect of confusing variables or time. In cases of the studies that implemented intervention in RTI approach, the first tier was coded as baseline, and the second or third tier intervention was coded as intervention phase for the analysis. Second, the scores on negative measures were reverse-coded. Third, multiple effect sizes from the same study or the same participant were avoided because of their dependence [45]. Thus, when there were multiple measurements regarding the same construct, the representative one was adopted or the mean score was calculated when using the same scale. In contrast, when dependent variables were corresponding to different academic domains (e.g., reading fluency and computational skills), multiple effect sizes were calculated independently.

## Results

### Characteristics of the studies

A total of 32 single-subject studies were selected for analysis. A total of 119 effect sizes from 1,601 data points and 100 participants were identified, which is summarized in Table 2.

**Table 2:** Number of participants and effect sizes.

Study	Number of participants (m,f)	Number of effect sizes
[63]	3 (0,3)	6
[64]	3 (1,2)	3
[65]	3 (3,0)	6
[66]	1 (0,1)	1
[67]	3 (2,1)	3
[68]	3 (3,0)	3
[69]	3 (1,2)	3
[70]	3 (3,0)	3
[71]	3	3
[72]	3 (1,2)	3
[73]	3 (1,2)	6
[74]	3 (3,0)	3
[75]	3 (2,1)	3
[76]	3 (2,1)	9
[77]	2 (1,1)	2
[78]	6	6
[79]	1 (0,1)	1
[80]	2 (1,1)	2
[81]	3 (3,0)	3
[82]	3 (2,1)	3
[83]	3 (3,0)	3
[84]	3 (2,1)	3
[85]	5 (3,2)	5
[86]	5 (2,3)	5
[87]	4 (2,2)	8
[88]	4 (3,1)	4
[89]	4 (2,2)	4
[90]	3 (1,2)	3
[91]	3 (2,1)	3
[92]	3 (3,0)	3
[93]	3 (2,1)	3
[94]	3 (2,1)	3
**Total**	**100**	**119**

Tables 3–6 present the characteristics of 32 single-subject studies in terms of general characteristics, participants-related characteristics, intervention-related characteristics, and DBI-related characteristics.

**Table 3:** General characteristics of the studies.

Category		N	%
**Year of publication**			
	2005–2009	6	18.8
	2010–2014	8	25.0
	2015–2020	18	56.3
**Type of publication**			
	Journal	14	43.8
	Dissertation	18	56.3
**Study design**			
	Multiple baseline	12	37.5
	AB	5	15.6
	ABA	3	9.4
	ABAB	1	3.1
	Alternating treatment	1	3.1
	Other	10	31.3
**Total**		32	100

**Table 4:** Participant-related characteristics of the studies.

Category			N	%	
**Grade level**					
	Elementary school	Low (1–3)	19	59.4	87.6
		High (4–6)	7	21.9	
		Low + High	2	6.3	
	Middle school		3	9.4	
	High school		1	3.1	
**Exceptionality type**					
	With or at risk of learning disability		9	28.1	
	Low achievement		2	6.3	
	Under achievement		14	43.8	
	Intellectual disability		5	15.6	
	Other		2	6.3	
**School type**					
	General classroom		20	62.5	
	Special education classroom		6	18.8	
	Special school		1	3.1	
	Mixed		3	9.4	
	Other		2	6.3	
**Total**			32	100	

**Table 5:** Intervention-related characteristics of the studies.

Category			N	%
**Number of sessions**				
	10–19		23	71.9
	20–29		7	21.9
	Above 30		2	6.3
**Number of sessions per week**				
	1		5	15.6
	2		25	78.1
	3		2	6.3
**Length of each session**				
	15–30 mins		2	6.3
	31–45 mins		23	71.9
	46–60 mins		6	18.8
	NA		1	3.1
**Duration**				
	4–9 wks		22	68.8
	10–14 wks		9	28.1
	15–19 wks		1	3.1
**Group size**				
	One-on-one		23	71.9
	Small (2–3)		3	9.4
	Medium (4–6)		2	6.3
	Mixed (One-on-one + group)		2	6.3
	NA		2	6.3
**Interventionist**				
	Researcher		24	75.0
	Graduate students		2	6.3
	Researcher + Volunteer		1	3.1
	Peer		1	3.1
	NA		4	12.5
**Instructional setting**				
	General classroom		16	50.0
	Special education classroom		3	9.4
	Special school		1	3.1
	Private center		1	3.1
	Other		5	15.6
	NA		6	18.8
**Basic academic domain (DV)**				
	Reading	Early literacy	1	3.1
		Reading fluency	7	21.9
		Fluency + comprehension	7	21.9
		Early literacy + Fluency + comprehension	1	3.1
	Writing		6	18.8
	Math	Early math	1	3.1
		Calculation	7	21.9
	Reading + Writing		2	6.3
**Total**			32	100

**Table 6:** DBI-related characteristics of the studies.

Category		N	%
**Type of CBM task (BASA)**			
	Early literacy	1	3.1
	Reading fluency	16	50.0
	Fluency + Comprehension	1	3.1
	Writing	6	18.8
	Early math	1	3.1
	Math	7	21.9
**Measurement frequency (monitoring)**			
	Every session	20	62.5
	Every 2–3 sessions	9	28.1
	Every 4 sessions	2	6.3
	Flexible	1	3.1
**Administrator of CBM**			
	Interventionist	24	75.0
	Other	2	6.3
	NA	6	18.8
**Data-based decision rules**			
	Slope	1	3.1
	Mastery criterion	6	18.8
	Slope + Data point	7	21.9
	Slope + Mastery	1	3.1
	Percentile	3	9.4
	Other	1	3.1
	NA	13	40.6
**Instructional change**			
	Quantitative	1	3.1
	Qualitative	6	18.8
	Quantitative + Qualitative	3	9.4
	NA	22	68.8
**Total**		32	100

### Analysis of quality indicators

Table 7 presents the results of QI ratings for single-subject design studies. It was interpreted as having a sufficient quality level when the indicators were rated 2 or more.

**Table 7:** QIs applied to single-subject design studies.

Dimensions	Specific indicators	Number of studies			M
		1 (unsatisfied)	2 (partially satisfied)	3 (satisfied)	
Participants / setting	participants characteristics (e.g., age, gender, disability, diagnosis)	0	4	28	2.88
	process for selecting participants	1	5	26	2.78
	information about interventionists or teachers and comparability across conditions	21	7	4	1.47
	critical features of the physical setting	9	17	6	1.91
Dependent variable	description of DV	0	1	31	2.97
	measurement process	0	0	32	3.00
	measurement validity and description	0	4	28	2.88
	measurement frequency	0	3	29	2.91
	data collected on reliability (e.g., IOA = 80%; Kappa = 60%)	22	0	10	1.63
Independent variable	description of IV	0	8	24	2.75
	IV manipulation	14	7	11	1.91
	fidelity of implementation	22	1	9	1.59
Baseline	DV measurement	3	11	18	2.47
	description of baseline condition	12	16	4	1.75
Experimental control/Internal validity	design demonstrates experimental effect	0	0	32	3.00
	design controls for common threats to internal validity (e.g., elimination of rival hypotheses)	12	7	13	2.03
	patterns of results	0	0	32	3.00
External validity	replication of effects across participants, settings, or materials	2	11	19	2.53
Social validity	social importance of DV	0	0	32	3.00
	social importance of magnitude of change in DV	0	12	20	2.63
	practicality and cost effectiveness of implementation of IV	22	5	5	1.47
	typical nature of implementation of IV	16	15	1	1.53

### Results from two-level meta-analysis

#### Results from the unconditional model: Mean effect size and heterogeneity

**Fig 2:**
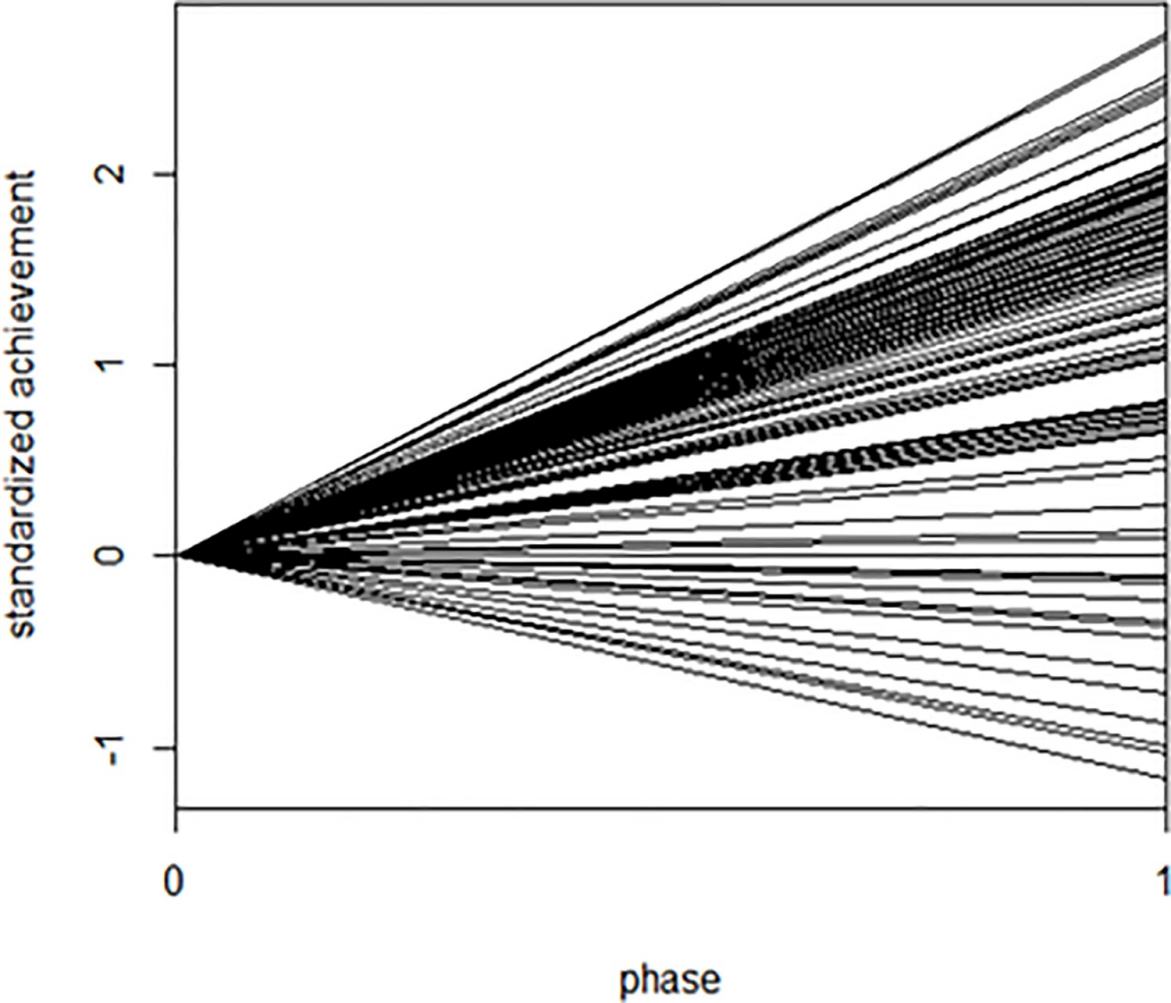
Effect sizes across participants.

The results from the unconditional model are displayed in Table 8. The mean of the effect sizes across all participants was 1.34. It was significantly different from zero, suggesting that DBI was effective in improving basic academic skills of participating school-aged students with learning difficulties. Additionally, the *χ*^2^ statistic accompanying these variance components indicated that there was significant variability between the effect sizes among 100 participants in their effect sizes, suggesting the need to identify the possible predictors. Fig 2 visually shows 119 effect sizes from 100 participants and the variability between those effect sizes. In Fig 2, 0 and 1 on the *x*-axis represent the baseline and intervention phase respectively; the *y*-axis represents the standardized data points of outcome variables; and the slope of each line indicates the standardized effect size of each dependent variable. Fig 2 also indicates that the effect sizes across participants were varying in a wide range.

**Table 8:** Results from the unconditional model.

**Fixed effect**	**Coefficient**	**SE**	***t***	***df***	***p*-Value**
Slope (*β*_10_)	1.341	0.078	17.224***	99	<0.001
**Random effect**	**SD**	**Variance component**	***χ*** ^**2**^	***df***	***p*-Value**
Slope (*γ*_1*j*_)	0.722	0.522	907.154***	99	<0.001
Level-1 error term (*ϵ*_*ij*_)	0.852	0.725			

*p < .05 **p < .01

***p < .001.

#### Mean effect size for each basic academic domain

Additionally, mean effect size for each basic academic domain (reading, writing, and math) was calculated. Phonological recognition, word recognition, reading fluency, and reading comprehension skills were included as outcome variables in reading. The studies included in writing were measuring the qualitative and quantitative scores, and the studies included in math were assessing early mathematics skills (e.g., number recognition, counting) and computational skills. The results of the unconditional model for each area are shown in Table 9. For reading, 916 data points from 60 participants were included in the analysis, accounting for the largest proportion. 586 data points from 24 study participants were identified in writing, and 275 data points from 22 participants were identified in math. The mean effect sizes for domains of reading, writing, and math, were 1.432, 1.520, and 1.091 respectively, and they were all significantly different from zero. Furthermore, in writing and math, there were significant variability across participants in effect sizes.

**Table 9:** Results from the unconditional model for reading, writing, and math.

**Reading**					
**Fixed effect**	**Coefficient**	**SE**	***t***	***df***	***p*-Value**
Slope (*β*_10_)	1.432	0.090	15.939***	59	<0.001
**Random effect**	**SD**	**Variance component**	***χ*** ^**2**^	***df***	***p*-Value**
Slope (*γ*_1*j*_)	0.633	0.400	429.965	59	>0.500
Level-1 error term (*ϵ*_*ij*_)	0.833	0.694			
**Writing**					
**Fixed effect**	**Coefficient**	**SE**	***t***	***df***	***p*-Value**
Slope (*β*_10_)	1.520	0.165	9.188***	23	<0.001
**Random effect**	**SD**	**Variance component**	***χ*** ^**2**^	***df***	***p*-Value**
Slope (*γ*_1*j*_)	0.782	0.611	370.814***	23	<0.001
Level-1 error term (*ϵ*_*ij*_)	0.802	0.643			
**Math**					
**Fixed effect**	**Coefficient**	**SE**	***t***	***df***	***p*-Value**
Slope (*β*_10_)	1.091	0.188	5.815***	21	<0.001
**Random effect**	**SD**	**Variance component**	***χ*** ^**2**^	***df***	***p*-Value**
Slope (*γ*_1*j*_)	0.823	0.678	208.508***	21	<0.001
Level-1 error term (*ϵ*_*ij*_)	0.847	0.718			

*p < .05 **p < .01

***p < .001.

#### Results from the conditional model: Impact of the predictors

Several variables related to participants, intervention, and DBI were added respectively to the two-level model as predictors in order to examine their impacts on the variability of effectiveness. Each variable was dichotomously coded for the analysis. For the missing data, the list-wise deletion was used. Table 10 shows the coding systems for the Level-2 predictors.

**Table 10:** Coding system for the Level-2 predictors.

Predictors		Coding	
		0	1
**Participant-related variables**			
	Grade level	Elementary school	Middle or high school
	Exceptionality type	with or at risk of learning disability	Without or not at risk of learning disability
		With low or under-achievement	Without low or under-achievement
		With intellectual disability	Without intellectual disability
	Gender	Male	Female
	School type	General classroom	Other than general classroom
**Intervention-related variables**			
	Number of sessions	19 sessions or less	More than 19 sessions
	Number of sessions per week	1	2–3
	Length of each session	45 minutes or less	More than 45 minutes
	Duration	9 weeks or less	More than 9 weeks
	Group size	One-on-one	Group
**DBI-related variables**			
	Measurement frequency for monitoring	Every session	Every 2 or more sessions
	Data-based decision rule	Including slope-based rule	Without including slope-based rule
	Instructional adaptation	Specified	Not specified

Table 11 shows the results from the HLM model that included the characteristics related to participants as Level-2 predictors. The results indicated that the presence of intellectual disability significantly accounted for the variance in effect sizes (*B* = 0.532, *p* = 0.019). To be specific, the effect size was 1.304 for students with intellectual disabilities, whereas the effect size was 1.836 for those with learning difficulties without intellectual disabilities (i.e., with or at risk of learning disabilities, low or under-achievement, etc.). However, the effect sizes did not vary as a function of the other variables. It can be interpreted that the effectiveness of DBI was significant regardless of the grade level, classification of learning disabilities, classification of low or under-achievement, gender, and school type.

**Table 11:** Results of the conditional model with participant-related variables as predictors.

Fixed effect		Coefficient	SE	*t*	*df*	*p*-Value
Grade level		0.043	0.161	0.267	79	0.790
Exceptionality type	Learning disability	-0.237	0.201	-1.184	79	0.240
	Low or under-achievement	-0.034	0.177	-0.195	79	0.846
	Intellectual disability	0.532	0.222	2.394*	79	0.019
Gender		0.352	0.177	1.988	79	0.050
School type		0.312	0.184	1.694	79	0.094

*p < .05 **p < .01 ***p < .001.

Table 12 shows the results from the model that included the intervention-related variables as Level-2 predictors. The number of sessions and the length of each session were the only predictors that significantly accounted for the variability in effect sizes across participants. Specifically, while the effect size was 1.552 when the intervention was provided less than 19 sessions, it was 0.984 when the intervention was continued for a longer period. In addition, whereas the effect size was 1.281 when the time per session was less than 45 minutes, it was 1.889 when each session was longer than 45 minutes. It was found that the effect sizes did not significantly vary as a function of the number of sessions per week, duration, and group size.

**Table 12:** Results of the conditional model with intervention-related variables as predictors.

Fixed effect	Coefficient	SE	*t*	*df*	*p*-Value
Number of sessions	-0.568	0.189	-3.001**	79	0.004
Number of sessions per week	-0.222	0.225	-0.989	79	0.325
Length of each session	0.608	0.212	2.870**	79	0.005
Duration	-0.193	0.189	-1.019	79	0.311
Group size	0.006	0.217	0.026	79	0.979

*p < .05

**p < .01 ***p < .001.

Table 13 presents the results from the model including DBI-related variables as predictors. The coefficients of the variables were not significant, which indicated that the variation of the effect sizes among participants was not explained by the variables related to the features of DBI. It can be interpreted that the effect of intervention based on DBI was significant regardless of the specific variables related to DBI. However, it should be noted that the information about DBI process was not sufficient in many studies.

**Table 13:** Results of the conditional model with DBI-related variables as predictors.

Fixed effect	Coefficient	SE	*t*	*df*	*p*-Value
Measurement frequency for monitoring	-0.164	0.185	-0.886	79	0.378
Data-based decision rule	-0.356	0.207	-1.714	79	0.090
Instructional adaptation	-0.301	0.194	-1.547	79	0.126

*p < .05 **p < .01 ***p < .001.

## Discussion

The purpose of this study was to identify the effectiveness of DBI for students with learning difficulties in Korea. Furthermore, the overall quality of the research that implemented data-based instruction for those students was determined in the study. The overall findings with respect to the research questions and directions for future research and practice were discussed below.

### Research trends on DBI

It was identified that research on DBI for students with learning difficulties have been conducted constantly in Korea since 2000s. It indicates that there has been a continuous need to support diverse students with learning difficulties and ongoing efforts to apply DBI for those students. The review of the studies revealed that most of the studies were conducted for elementary school students in general classroom. Reading fluency was set as the outcome variable with the highest ratio. It is consistent with the previous findings that more than 80% of students with learning disabilities have difficulties in reading [46] and that reading fluency is a highly reliable indicator of the overall reading abilities, encompassing several linguistic skills in it [47–49]. As for the characteristics of intervention, the results reveal that the most studies consisted of a total of 10 to 19 sessions, twice a week, and 31 to 45 minutes per session. Regarding characteristics of DBI, CBM for reading fluency was most frequently used, and students’ progress was measured every session in the most studies. Additionally, the slope-based method and the point-based method were mostly used together, and instructional adaption was mostly conducted in qualitative aspects.

Despite these efforts to implement DBI in research and practice, it was identified that many studies did not report sufficient information about the principles or procedures of DBI in a systematic way. For instance, about half of the studies did not provide specific information on instructional change and data-based decision rules. It was revealed in the previous finding that the frequency and quality of instructional changes have significant impact on the students’ achievement [10]. It not only benefits the students with learning difficulties but also teachers teaching them. For instance, in the study of McMaster et al. [15], teachers who conducted DBI made instructional adaptions more frequently in more various aspects, specifically based on convincing data rather than their intuition. Thus, future research and practice are needed to follow the procedures of DBI more systematically and to present the related information sufficiently.

### Quality of research on DBI

The overall quality of the studies was quite high. To be specific, 14 out of 22 Qis in single-subject design were rated as 2 points or more. Moreover, except for two, all studies scored an average of 2 points or higher. It indicates that research on DBI for school-aged student with learning difficulties have a sufficient quality in terms of research design and method. However, there were a few indicators scored below 2, which can be considered systematic weaknesses across studies. Specifically, in the future research, indicators regarding description of interventionist, comparability of interventionists across conditions, fidelity of intervention, manipulation of independent variable, and reliability of data collection are especially needed to be reported explicitly for the more reliable results.

### Effectiveness of DBI

The effectiveness of DBI for school-aged students with learning difficulties was found to be quite high. The mean effect size from single-subject design studies was 1.34 and statistically significant. Although it cannot be directly compared with the effect sizes extracted from the meta-analysis of group design studies, it reveals that DBI is quite effective to improve basic academic skills of students with learning difficulties. It was suggested that students with learning difficulties tend to acquire and develop their learning skills at a slower pace than their peers [50, 51]. It was also reported that for those students, intensifying intervention in a generic way is sometimes not enough to increase their achievement [5, 52]. Considering these previous findings, the result of the present study indicates that DBI is highly effective and suitable for those students. In addition, the use of CBM in DBI for assessing their achievement and growth seems to account for this large effectiveness. According to the previous finding, CBM tools can measure the ability and growth of students with learning difficulties in a more sensitive way [53]. For instance, CBM in reading measures the reading abilities with texts that are equivalent and comparatively easy-to-read across different grades [18]. Thus, the large effectiveness of DBI can be explained by the use of adequate measure for students with learning difficulties, and it should be noted that it is not only difficult but also unfair sometimes to check the progress of struggling learners with the identical measure used for those without learning difficulties.

### Variability across participants in effect sizes

The analysis of single-subject design studies revealed that there was significant variance across participants in effect sizes. The results from the conditional model showed that exceptionality type, the number of sessions, and the length of each session were significantly accountable for the variability of effect sizes.

Regarding the difference according to the presence of intellectual disabilities, the cognitive characteristics of students with intellectual disabilities seem to be associated to the comparatively lower effect size. In the present study, the number of participants with intellectual disabilities was 14, and the number of effect sizes was 17 in total. The average of their IQ was 55.9, which is within the range of mild intellectual disability. The previous findings suggested that students with mild intellectual disabilities have educational needs distinguished from other students because of their cognitive features [54, 55]. However, since the number of effect sizes was relatively small, it would require careful interpretation. Furthermore, it should be rather emphasized that the effectiveness of DBI for students with intellectual disabilities was significant as well and still was quite high (ES = 1.304). It means that DBI is quite effective and applicable for students with intellectual abilities as well as students without disabilities. Snyder & Ayres [56] suggested that widely used measurement for basic academic skills such as the Woodcock Reading Mastery Test (WRMT) and Comprehensive Test of Phonological Processing (CTOPP-2) are not suitable for those students due to its difficulty and unfamiliarity. In contrast, the general outcome measures such as CBM are regarded as more appropriate to evaluate the reading level and progress of students with intellectual disabilities [57]. Moreover, CBM is also recommended when making IEP goals for students with intellectual disabilities in the areas of reading, writing, and mathematics [58]. Thus, consistent with the previous findings, the results of the present study indicate that DBI and CBM can be applied for teaching students with intellectual disabilities.

Next, regarding the variance resulted from the number of sessions and the length of each session, more research is needed in the future. There are some inconsistent findings related to what is the most effective duration and length of sessions for DBI. For example, in the study of McMaster et al. [15], where conducted DBI in writing for 20 weeks, recommended that teachers conduct interventions at least three times a week, 20∼30 minutes per session. In another study, the total number of weeks of DBI was found to significantly predict the improvement of students’ progress [59]. Therefore, further research on the optimal duration of intervention and time per session in DBI is needed.

### Need of teacher support for DBI in Korea

When implementing DBI, the provision of support for teachers has been constantly suggested as the variable that significantly increases its effectiveness [8, 15]. However, there were very few research that provided teacher support. In the study of Choi & Kwon [60], it was revealed that many teachers in Korea were struggling with difficulties due to the lack of knowledge and information about instructional adaptations. Therefore, it is required to provide understandable information and constant support for special and general teachers for implementing DBI. For instance, What Works Clearninghouse (WWC) platform from the U.S. Department of Education, which allows teachers to search, compare, and evaluate DBI for each basic academic area, and National Center on Intensive Intervention (NCII), which provides a tool chart and a checklist for monitoring progress and changing instruction, could be referred [61].

### Study limitations

Findings of the present study should be interpreted in light of the following limitations. First, although the studies which met the selection criteria were included in the analysis, sufficient information related to DBI such as data-based decision rules and instructional changes were missing in many of them. Thus, there was a limit to analyze the impact of the probable predictors on variance in the effect sizes. Future study is needed to include more detailed information about DBI process, and there is need to analyze and compare the moderating effect of diverse variables. Second, two-level meta-analysis instead of three-level meta-analysis was implemented to synthesize the effect from single-subject design studies. It is recommended to conduct three-level meta-analysis of single-subject studies when there are more studies accumulated. Third, for calculating and synthesizing the effect sizes from single-subject design studies, the statistical method suggested by Van den Noortgate & Onghena [32–34] and applied by Wang, Cui, & Parrila [35], Wang, Parilla, & Cui [36], and Heyvaert et al. [37] was used. However, since there is no consensus yet for synthesizing the results of single-subject studies, it is necessary to calculate and compare the effect sizes calculated from various statistical methods. Moreover, future study is needed that considers the variability of the students’ previous achievement levels more in depth when interpreting the effect sizes. Fourth, in this study, only studies which used BASA as CBM tools were included for the analysis. It was because BASA was the standardized CBM widely used in basic academic areas in Korea. However, there is need that more various CBM tools with high validity and reliability should be developed and standardized in Korea. Then, future study is needed to synthesize the effectiveness and quality of DBI research using various CBM tools. Fifth, the mean effect size of DBI for each basic academic area was identified, but the difference in effectiveness depending on the specific focus of intervention or strategies was not analyzed in this study. Therefore, in the future study, the difference in effect sizes of DBI as function of specific instructional methods or contents should be examined. Sixth, in the present study, the QIs of Horner et al. [25] were used to evaluate the quality of individual studies. However, there is still need to examine the quality of the studies based on various criteria, especially beyond the aspects of research designs or methods. For example, Shin [61] argued that the criteria suggested by Fuchs, Fuchs, & Malone [62] and NCII, which reflect perspectives of teachers, should be considered when evaluating the quality of DBI.

## Supporting information

S1 Checklist PRISMA 2009 checklist. (DOCX)

S1 File (DOCX)
