# Supplementary material for: The Effects of Data-based Instruction (DBI) for Students with Learning Difficulties in Korea: A Single-subject Meta-analysis
Source: PLoS One. 2021 Dec 23;16(12):e0261120. doi: 10.1371/journal.pone.0261120 (PMC8699614; doi:10.1371/journal.pone.0261120)
Supplement: S1 File — (DOCX) [file pone.0261120.s002.docx]

1. Shim MO, Park HO. The effect of E-NIE program on the reading fluency and writing of a student with intellectual disabilities. The Journal of Developmental Disabilities. 2015;19(3): 123-146.
2. Kwon HJ. The effects of cognitive processing training based on PASS Reading Enhancement Program (PREP) on the reading speed and accuracy of the student with mild mental retardation [master’s thesis]. Kongju National University; 2014.
3. Cho GH. The effects of Readers Theater program on reading fluency and reading comprehension of reading underachievers [master’s thesis]. Seoul National University; 2008.
4. Choi JK. A study on the meaning of goal setting and effects of intervention within curriculum-based measurement. Asian Journal of Education. 2008;9(2): 89-112.
5. You EK. The effects of national language differentiated instruction on reading ability of underachievers in national language [master’s thesis]. Daegu National University of Education; 2016.
6. Kim KH. The effects of children’s poem program based on the balanced approach to language education on reading skills of the underachieved students in Korean language. [master’s thesis]. Daegu National University of Education; 2017.
7. Lee HJ, Lee TS. The effects of language experience approach utilizing graphic organizer on writing abilities of students with mild intellectual disabilities. Journal of Special Education. 2019;26(1): 87-108.
8. Kwon DY, Kang OR. The effects of mathematical learning through the play activities on computation abilities and mathematical attitudes of low-achieving students. The Korea Journal of Learning Disabilities. 2007;4(2): 71-91.
9. Yu JW. Effects of written expression on written ability and fluency for students with learning difficulties in middle school [master’s thesis]. Chonnam National University of Education; 2010.
10. Han YS. Effects of book discussion activities on writing achievements for at-risk students with learning disabilities in writing [master’s thesis]. Daegu National University of Education; 2014.
11. Park SH, Lee KJ. Effects of whole language approach with fairy tales on reading abilities of students with learning difficulties. The Journal of Korea Elementary Education. 2015;26(2). 231-245.
12. Lee EO. A study on the effects of writing book reports using a peer tutoring writing skills improvement of the student with severe learning disability [master’s thesis]. Chonnam National University of Education; 2015.
13. Kwon MY. Effects of the mindfulness-based reading intervention on the attention behavior and reading fluency of students with learning disabilities. Journal of Special Education & Rehabilitation Science. 2015;52(2): 1-29.
14. Shin SJ, Kang OR. The effects of synthetic phonics training based on explicit instruction on word recognition, oral reading fluency and spelling of students at risk for dyslexia. The Korea Journal of Learning Disabilities. 2018;15(3), 103-134.
15. Kim DI, Koh HJ, Yi HL. BASA implementation for children with learning disabilities: The one-year CBM case study in Korea. The Journal of Special Education: Theory and Practice. 2014;15(1): 193-213.
16. Kim BR. The effects of RTI as an intervention and diagnostic approach for at-risk students in mathematics [master’s thesis]. Gyeongin National University of Education; 2011.
17. Ko YS, Song JH. A case study on learning consulting for children with poor mathematics. Journal of School Psychology and Learning Consultation. 2019;6(1): 1-25.
18. Kim MJ, Lee JW, Lee DC. The effect of reading guidance through smart learning on reading fluency of students with intellectual disability. Journal of Special Education. 2014;21(2): 196-220.
19. Lee JY. The effect of story retelling activities on reading abilities of ADHD-risk students [master’s thesis]. Daegu National University of Education; 2015.
20. Kim MK, Lee KJ. Effects of reverse-role tutoring on reading fluency and academic self-confidency of at-risk readers. The Korea Journal of Learning Disabilities. 2015;12(2); 185-201.
21. Gil YM. The effect of collaborative writing with classical literature on writing achievements for underachievers in Korean language [master’s thesis]. Daegu National University of Education; 2015.
22. Seul YG. A study on change in reading abilities of children with reading disabilities through interested reading programs [master’s thesis]. Yosu National University; 2005.
23. Kim DI, Shin HYG, Kim HJ, Cho EJ. An analysis of the effects of the synthetic phonics program on phonological awareness skills of underachieving children. The Journal of Special Children Education. 2018;20(2): 25-50.
24. Hwang AH. The effects of teaching phonics through direct instruction and fluency training on reading for poor readers [master’s thesis]. Duksung Women’s University; 2012.
25. Ahn SJ. The effect of scaffolding intervention program to improve reading achievement in elementary students with poor reading [master’s thesis]. Seoul National University; 2018.
26. Choi EJ. The effects of reading strategy training on reading disorder children’s reading ability and reading attitude [master’s thesis]. Daegu University; 2006.
27. Jung KJ, Lee HJ. The study on the applicability of response to intervention. Korean Journal of Special Education. 2009;44(2): 313-339.
28. Song PR, Kim DI. The effects of subitizing based numbers sense intervention on number sense and counting for students at-risk of MLD. The Korea Journal of Learning Disabilities. 2020;17(1), 127-157.
29. Kim ES. Applicability research of direct instruction addition and subtraction program for mathematical underachievers in elementary school [master’s thesis]. Seoul National University; 2015.
30. An MS. The effects of computer assisted instruction on the addition and subtraction computation performance of middle school students with intellectual disabilities [master’s thesis]. Dankook University; 2012.
31. Jeong CM. The effect of creative summary note on writing achievements for underachievers in Korean language [master’s thesis]. Daegu National University of Education; 2015.
32. Kim DI, An YJ, Cho EJ, Choi SH. A case study of individualized reading fluency program for students at-risk of learning disabilities. Journal of Research in Curriculum & Instruction. 2020;24(6): 589-601.
